# Synthetic bacterial stem cells and their multicellularity for synthetic biology and beyond

**DOI:** 10.1093/synbio/ysz023

**Published:** 2019-08-28

**Authors:** Daniel Bojar

**Affiliations:** 4058 Basel, Switzerland

Differentiation produces the plethora of different cell types in any multicellular organism. One of the core principles allowing stem cells to produce differentiated daughter cells is asymmetric cell division, creating two cells with different cellular content (1). The transcription factors and signaling complexes which remain in one of these cells then commit it to differentiate into a specific lineage whereas the other daughter cell replenishes the stem cell population.

Being a paradigm of multicellular eukaryotic organisms, asymmetric cell division to kickstart differentiation is largely absent in bacteria and prokaryotes in general. In a recent publication, the group around Matthew R. Bennett at Rice University (with Sara Molinari and David L. Shis in the lead) described the introduction of asymmetric cell division into the bacterium *Escherichia coli* using principles from synthetic biology (2). Implementing guiding ideas from engineering into biology, synthetic biology aims to modify biological systems in a rational and predictable manner, mainly through genetic modification.

One of these engineering principles is the usage of modular parts when constructing a system. Bennett and colleagues used the chromosome partitioning system of another bacterium, *Caulobacter crescentus*, as a unit in their design. Consisting of the DNA-binding protein ParB and the DNA element *parS*, the chromosome partitioning system is modular enough to be transferable to *E. coli*. Integrating the *parS* DNA sequence into a plasmid which additionally carries a gene expression cassette, for instance for a fluorescent protein, then causes ParB to bind to the *parS* element. Forming a cluster, *parS*-containing plasmid is then exclusively and asymmetrically present in one of the two daughter cells after the process of cell division.

And now here comes the trick: by making the production of ParB contingent on the presence of a small molecule (for instance by using arabinose-inducible promoters), the researchers can control when exactly they want to initiate asymmetric cell division. This way, a sustainable population of stem cell-like bacterial cells containing the *parS*-marked plasmid can be replenished at every cell division event, spawning descendant, differentiated cells in the process.

To further build on their approach, the Bennett lab then added an orthogonal chromosome partitioning system (this time consisting of the DNA-sequestering SopB and the DNA-element *sopC* from the F plasmid of *E. coli*). Controlled by a different small molecule, isopropyl β-d-1-thiogalactopyranoside, their final system now had three differentiated and one pluripotent stem cell-like state. Adding each of the inducers (or both) led to distinct differentiated states, differing in the gene-carrying plasmids with the *parS* or *sopC* DNA elements.

Establishing a bacterial stem cell population able to differentiate into multiple states, this publication lays the groundwork for prokaryotic multicellular organisms, which will require multiple cell types harboring different functionalities. This bottom-up design approach might reveal new insights into the requirements or characteristics of multicellular arrangements. Additionally, by combining several specialized cell populations, such an approach is also certain to substantially enhance the potentialities of prokaryotic synthetic biology endeavors, ranging from biofactories to therapeutic assemblies.

Yet, of course, the presented systems still have some drawbacks and room to improve. The possibility to differentiate solely by losing a plasmid makes design more difficult and indirect, as inhibition needs to be removed for an activation to occur upon differentiation. Thus, the addition of a mechanism for adding genes or plasmids in the process of differentiation would greatly enrich synthetic bacterial stem cells. It will also be interesting to see what kind of functionalities benefit in practice from the multicellular arrangement presented by the authors, which has to be established in future work.
